# Detection of Vaccine-Induced Antibodies to Ebola Virus in Oral Fluid

**DOI:** 10.1093/ofid/ofw031

**Published:** 2016-02-22

**Authors:** Teresa Lambe, Tommy Rampling, Dhan Samuel, Georgina Bowyer, Katie J. Ewer, Navin Venkatraman, Matthew Edmans, Steve Dicks, Adrian V. S. Hill, Richard S. Tedder, Sarah C. Gilbert

**Affiliations:** 1 Jenner Institute, University of Oxford; 2 Virus Reference Department, National Infection Service, Public Health England; 3 Transfusion Microbiology, National Health Service Blood and Transport, Colindale, London, United Kingdom

**Keywords:** antibodies, Ebola vaccine, oral fluid, serology

## Abstract

Blood sampling to assess production of antigen-specific antibodies after immunization is commonly performed, but it presents logistical difficulties for trials carried out during an infectious disease outbreak. In this study, we show that antibodies may be reliably detected in oral fluid collected in a minimally invasive manner without use of sharps.

**Clinical Trials Registration.** NCT02240875.

As part of the global effort to end the 2014 outbreak of Ebola virus disease in Guinea, Sierra Leone, and Liberia, clinical trials of novel vaccines against Ebola were fast tracked. The most advanced vaccines in clinical development are the replication-competent vesicular stomatitis virus (VSV)-vectored vaccine, and the replication-deficient simian adenovirus ChAd3-vectored vaccine. The VSV-vectored vaccine results in seroconversion in all vaccinees 28 days after immunization in phase I trials [1] and was the first to be tested in an efficacy trial using ring vaccine in Guinea, demonstrating remarkably high efficacy [2]. The ChAd3-vectored vaccine also results in seroconversion with the antibody response peaking 28 days after immunization, although this response is detectable in many vaccinees after 14 days [3, 4]. Other vaccines are also in clinical development. Phase I and II trials to assess vaccine safety and immunogenicity have taken place in the United States, United Kingdom, Switzerland, Germany, China, Mali, Uganda, Kenya, Gabon, Ghana, Sierra Leone, Senegal, Australia, and Liberia among others [2, 5].

The quantification of antibodies in serum samples is a standard method to assess a response to vaccines, but it requires invasive sampling to be conducted by a trained operator, and the use of needles in these cases carries a risk of needlestick injuries. An alternative method is to collect oral fluid (ORF) as opposed to saliva, sometimes described as oral mucosal transudate, from the gingival crevice (between teeth and gums) using a swab and to assess antibody levels in the ORF. The level of immunoglobulin (Ig)G in ORF is considerably higher than that in saliva [6]. In earlier studies, ORF has been used to detect antibodies to viral infections, including mumps, measles, and rubella [7], and routinely used for the diagnosis of hepatitis A. This method was tested during the EBL01 trial [4] in Oxford, United Kingdom, and Ebola-specific antibody responses in ORF and serum samples taken on the same day were compared.

## MATERIALS AND METHODS

### Oral Fluid and Serum Samples

Trial registration and volunteer recruitment for the ChAd3-priming vaccination has been described previously [4]. At enrollment, 76 subjects received a single priming immunization with ChAd3 EBO-Z at either 1 × 10^10^ virus particles (vp) (n = 20), 2.5 × 10^10^ vp (n = 36), or 5 × 10^10^ vp (n = 20). Forty-six of these subjects then received a single boosting immunization with MVA-BN Filo at either 1.5 × 10^8^ plaque-forming units (pfu) (n = 34) or 3 × 10^8^ pfu (n = 12) (K. E., unpublished data). Boosting immunization was given at an interval of between 1 and 10 weeks after priming immunization. Safety follow-up visits were conducted at various time points up to 6 months after boosting immunization. Blood was collected for Ebola glycoprotein (GP)-specific antibody titers at immunization and during safety follow-up visits. Oral fluid was collected using Oracol swabs (produced by Malvern Medical Developments Limited) at immunization and during safety follow-up visits. It was collected by rubbing the sponge swab firmly along the gum at the base of the teeth for approximately 1 minute. Swabs were then placed in the supplied test tube, returned to the laboratory without refrigeration, and stored for up to 7 days at 4^°^C before adding 1 mL of transport medium (TM) to the tube. The swab was agitated through the TM, eluted as described [8], and the eluate was stored for further testing.

### Enzyme-Linked Immunosorbent Assay

Standardized indirect IgG enzyme-linked immunosorbent assays (ELISAs) were conducted using plates coated with Zaire strain Ebola GP. Processed ORF eluates were added directly to the plate. Serum samples were added at dilutions of 1:100 or occasionally 1:500. The standard curve method [9] was used with serial dilutions of a reference pool of Zaire GP-positive sera that was included on all plates. The standard curve was graphed using a 4-parameter curve, and an arbitrary number of ELISA units were assigned to individual points in the dilution series, thus facilitating the calculation of arbitrary ELISA units for each unknown sample. A blank containing only assay diluent was used to correct for background, and in all experiments it had an absorbance value of less than the maximum of 0.15.

### Immunoglobulin IgG or IgM Capture Assay

In brief, 96-well microtiter plates were coated with rabbit or goat antibody to human ɣ or µ Fc, respectively. Sera diluted at 1:800 or ORF eluates undiluted were incubated in the coated wells, which captured the respective Igs from the donor sample. ZEBOV GP antigen (rGPδTM; IBT Bioservices Inc.) conjugated to horseradish peroxidase was added to the wells, and if specific antibody was captured by the solid phase it was able to bind and was revealed by the addition of tetramethylbenzidine TMB substrate. A human convalescent plasma (UK1) and pooled normal human plasma provided controls, and the cutoff was the mean of 4 negative replicates plus an optical density of 0.1.

## RESULTS AND DISCUSSION

**Figure 1.**
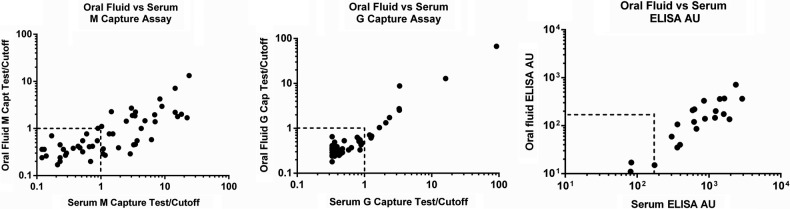
Comparison of antibody levels in paired oral fluid and serum from participants in the EBL001 clinical trial. (A) Immunoglobulin (Ig)M antibody levels correlated well between paired oral fluid and serum as assessed with an IgM capture assay (*r* = 0.78, *P* < .0001, 2-tailed nonparametric Spearman's correlation). (B) Immunoglobulin G antibody levels correlated well between paired oral fluid and serum as assessed with an IgG capture assay (*r* = 0.68, *P* < .0001, 2-tailed nonparametric Spearman's correlation). (C) IgG antibody levels correlated well between paired oral fluid and serum as assessed with a standardized enzyme-linked immunosorbent assay (*r* = 0.83, *P* < .0001, 2-tailed nonparametric Spearman's correlation). Dashed lines indicate assay-dependent cutoff point for sample positivity.

Participants in the EBL01 clinical trial, which tested the use of ChAd3 priming followed by MVA boosting ([4]; K. E., unpublished data), were requested to provide ORF samples at the same time as blood samples, in a pilot study of this sampling method. To assess a range of expected antibody titers, samples were taken after ChAd3 prime only or after ChAd3 prime and MVA boost. Sample collection was well accepted by clinical trial participants and took <5 minutes per person. Antibodies in ORF and the paired serum sample were compared using a variety of assays. Figure 1 shows a strong correlation between assays conducted on paired ORF and serum samples using either an indirect ELISA, an IgG capture assay, or an IgM capture assay. The detection of IgM class antibody in serum and ORF provides for the detection of the early immune response, on occasion before detectable IgG, and will enable identification of recent infections in field studies.

The results of the EBL01 trial have been reported elsewhere ([4]; K. E., unpublished data). Antibody responses were detected in serum after ChAd3 priming and were substantially increased after MVA boosting. Using each of the 3 assays used to quantify antigen-specific responses described here, there was a strong correlation between response detected in the serum and the paired ORF sample. The ability to detect the presence of Ebola GP-specific antibody at low titer is chiefly determined by the assay used, the volume of TM used to elute the ORF from the swab, and the extent of sample dilution. For the IgG and IgM capture assays, which rely on measuring the proportion of antigen-specific antibody captured onto the solid phase and not the concentration of antigen-specific antibody present [6], the test/cutoff signals generated in these assays for serum and matched ORF samples often correlate well, provided the concentration of total antibody, both specific and nonspecific, is sufficient to saturate the solid phase. The test/cutoff for undiluted ORF eluates was equivalent to on average 80% of that of matched sera diluted 1:800 in the capture assays. In the indirect ELISA assay, the standardized antibody titer in ORF eluates was equivalent to, on average, 20% of the titer of matched sera. This sample collection method is well suited to the assessment of antibody responses in clinical trials of vaccines, and it could be used for follow up of vaccinees to study duration of antibody response without the need for invasive sampling or related training and thereby removing the risk of needlestick injury. It could also provide a suitable community sampling and testing protocol for field studies defining seroprevalence of antibodies after EBOV infection. This may be useful for assessing public health interventions.

## CONCLUSIONS

The choice of assay to measure vaccine-induced antibodies will depend on whether a standardized assay is available, and, if not, it would be preferable to use the same assay that had been used during earlier clinical trials of the vaccine under development to allow comparison with data generated during those trials [4]. In this pilot study, there was a high correlation between vaccine-induced antibodies to Ebola Zaire GP using 3 different assays over a wide range of titers, and this sampling method should be considered for use in clinical trials of novel vaccines especially when these are conducted during an infectious disease outbreak.
